# Key elements in assessing the educational environment: where is the theory?

**DOI:** 10.1007/s10459-011-9346-8

**Published:** 2012-02-04

**Authors:** Johanna Schönrock-Adema, Tineke Bouwkamp-Timmer, Elisabeth A. van Hell, Janke Cohen-Schotanus

**Affiliations:** Center for Research and Innovation in Medical Education, University of Groningen & University Medical Center Groningen, Antonius Deusinglaan 1, 9713 AV Groningen, The Netherlands

**Keywords:** Educational environment, Instrument development, Learning environment, Medical education, Theoretical framework

## Abstract

The educational environment has been increasingly acknowledged as vital for high-quality medical education. As a result, several instruments have been developed to measure medical educational environment quality. However, there appears to be no consensus about which concepts should be measured. The absence of a theoretical framework may explain this lack of consensus. Therefore, we aimed to (1) find a comprehensive theoretical framework defining the essential concepts, and (2) test its applicability. An initial review of the medical educational environment literature indicated that such frameworks are lacking. Therefore, we chose an alternative approach to lead us to relevant frameworks from outside the medical educational field; that is, we applied a snowballing technique to find educational environment instruments used to build the contents of the medical ones and investigated their theoretical underpinnings (*Study 1*). We found two frameworks, one of which was described as incomplete and one of which defines three domains as the key elements of human environments (*personal development/goal direction*, *relationships*, and *system maintenance and system change*) and has been validated in different contexts. To test its applicability, we investigated whether the items of nine medical educational environment instruments could be mapped unto the framework (*Study 2*). Of 374 items, 94% could: 256 (68%) pertained to a single domain, 94 (25%) to more than one domain. In our context, these domains were found to concern *goal orientation*, *relationships* and *organization/regulation*. We conclude that this framework is applicable and comprehensive, and recommend using it as theoretical underpinning for medical educational environment measures.

## Introduction

The educational environment has been increasingly acknowledged as vital for high-quality medical education (Roff 2005; WFME 2003, 2007; Genn 2001a, b). Important components of the educational environment include atmosphere, number of (formal) learning opportunities and available facilities. The value of the educational environment for the quality of education is underpinned by research outcomes, showing that students’ perceptions of the educational environment quality influence their involvement, satisfaction and success (De Young 1977; Haertel et al. 1981; Karagiannopoulou and Christodoulides 2005; Müller and Louw 2004). For example, a positive educational environment is a necessary condition to motivate student learning (Kirkpatrick 1996; Müller and Louw 2004). In medical education, the growing acknowledgement that a positive environment contributes to the quality of education has stimulated the development of several educational environment instruments (Bloomfield and Subramaniam 2008; Cassar 2004; Holt and Roff 2004; Mulrooney 2005; Roff et al. 1997, 2005; Rotem et al. 1995).

Examination of recent medical educational environment instruments reveals that there are many differences between them. These differences are in part attributable to the fact that the instruments are often tailored to a specific setting of interest (Bloomfield and Subramaniam 2008; Cassar 2004; Holt and Roff 2004; Mulrooney 2005; Roff et al. 2005; Rotem et al. 1996). However, even though differences between settings may call for some tailoring of instrument content (Holt and Roff 2004; Patel and Dauphinee 1985), the array of differences is not restricted to item formulation: it also concerns instrument structure (i.e. the organization of items in scales) and scale names. From these differences, we gather that up till now there is no consensus about which concepts should be measured to ascertain the quality of the medical educational environment adequately.

From the publications on the development processes of the medical educational environment instruments, we noticed that the majority of them were not based on theory. The absence of a theoretical framework may explain the differences regarding the concepts measured. Having such a framework might help us to construct instruments that cover the entire educational environment and measure the essential concepts. As a result, educational environment quality might be measured more adequately. Therefore, the aims of this study were (1) to find a comprehensive theoretical framework that outlines the key concepts that should be measured to ascertain the quality of the educational environment, and (2) to test the applicability of this framework. We hoped such a framework would help us to answer two important questions. First, which concepts should be measured? Second, do medical educational environment instruments measure these essential concepts?

Based on our observation that the majority of the medical educational environment instruments were not founded on a theoretical framework and given the value and importance that is increasingly attached to the use and explicit formulation of a theoretical framework (Prideaux and Bligh 2002; Eva 2008; Eva and Lingard 2008; Bordage 2009), we wondered whether there are any theoretical frameworks that specify which elements of the medical educational environment should be measured to obtain an adequate and complete picture of its quality. Therefore, we originally conducted a systematic review of the literature to find theoretical frameworks that define which key concepts should be measured to ascertain the quality of the medical educational environment. However, our search of medical education databases and of educational and psychological databases did not yield any generally accepted theoretical frameworks. In a further attempt to find a theoretical framework, we chose a different approach to lead us to relevant frameworks from outside the medical field: using a snowballing technique (Teunissen and Westerman 2011), we tried to ascertain which educational environment instruments were used to build the contents of the medical ones. We then explored the descriptions of the developments of these underlying instruments to find out which theoretical frameworks, if any, were used to build the contents of these instruments (*Study 1*). To test the applicability of potentially relevant theoretical frameworks for medical education, we investigated whether the contents of available medical educational environment instruments corresponded with the framework (*Study 2*). The methods and results sections of each study are described ‘en bloc’.

*Study 1: In-depth search for theoretical frameworks*

## Methods

Following the Cochrane Library and the Best Evidence Medical Education (BEME) guidelines, we performed a thorough search for articles describing the development of medical educational environment instruments (Moher et al. 2009; Best Evidence Medical Education Collaboration 2003). Starting with the included publications, we applied a snowballing technique—comparable to the method applied by Teunissen and Westerman (2011)—in order to find a theoretical framework, which defines the essential concepts that should be measured to ascertain the quality of the educational environment. The first step was to explore whether the medical educational environment instruments were constructed using previously developed educational environment instruments. To ease the readability of our text, we will call the latter *underlying* instruments. The second step involved the examination of which theoretical frameworks, if any, were used to construct these underlying instruments. Subsequently, we repeated step 1 and 2 for all underlying instruments until arriving at their origins.

### Data sources and selection of instruments

Our search plan included electronic and hand searching. We systematically searched seven databases for relevant publications describing the development of medical educational environment instruments: Academic Search Premier, CINAHL, EMBASE, ERIC, MEDLINE, PsycARTICLES and PsycINFO. The search was performed in May 2011. The keywords used were

- develop*, construct*, devis*, devic* or design* in title or in abstract;
- survey, test, scale, measure, instrument, inventory or questionnaire in title or abstract;
- ‘learning environment’ or ‘education* environment’ in title or abstract; and
- ‘medical education’, ‘medical school’ or ‘medical training’ in text.

We limited our searches to publications in English. The first author performed the literature search.

We included studies that were readily accessible through the library or via internet, or that could be obtained through personal contact, if they met the following inclusion criteria:

- the *principal* aim of the study was to *develop* an instrument
- for *medical* education
- measuring the *educational* environment.

We excluded

- studies in which the development of an educational environment instrument only implied abbreviating an existing instrument;
- studies focusing on educational settings other than the medical education setting, for example nursing or dentistry;
- (narrow) studies focusing on specific aspects of the educational environment;
- (extensive) studies in which the educational environment was not the main focus of the instrument constructed;
- paper and poster abstracts, proceedings of conferences, dissertation abstracts, editorials and letters.

Two authors decided on the basis of title and abstract whether the inclusion criteria were met. They discarded irrelevant citations and evaluated the full text articles of the remaining citations for eligibility. Disagreements and uncertainties were resolved by consulting the other authors. We supplemented our search with relevant publications from the reference lists of publications identified as eligible. To ensure that our search was comprehensive at the time that we submitted our manuscript, we supplemented our electronic search with a manual search of four leading medical education journals: Medical Education, Academic Medicine, Advances in Health Sciences Education and Medical Teacher. We scanned the tables of contents in the issues from January through December 2011 and inspected early online releases. Conform our electronic search, we checked the abstracts of potentially relevant articles and—upon inclusion—we also checked their reference lists.

### Data abstraction

We ascertained which educational environment instruments were used to build the medical ones that were included in our study and retrieved the publications describing the development processes of these underlying instruments. We examined these publications to find out whether these instruments were founded on any theoretical frameworks. We repeated this process for any previously developed educational environment instruments that were used to construct them. We evaluated the usefulness of any theoretical frameworks found. We emanated from the point of view that—in order to be acceptable as a theoretical framework—frameworks should cover the entire environment, have been tested repeatedly and be generally acknowledged rather than only being a speculative view or idea (Rees and Monrouxe 2010). We considered a theoretical framework relevant and useful if it clearly delineates which components of the educational environment should be measured in order to obtain an adequate and complete picture of its quality.

## Results

### Search results

**Fig. 1:**
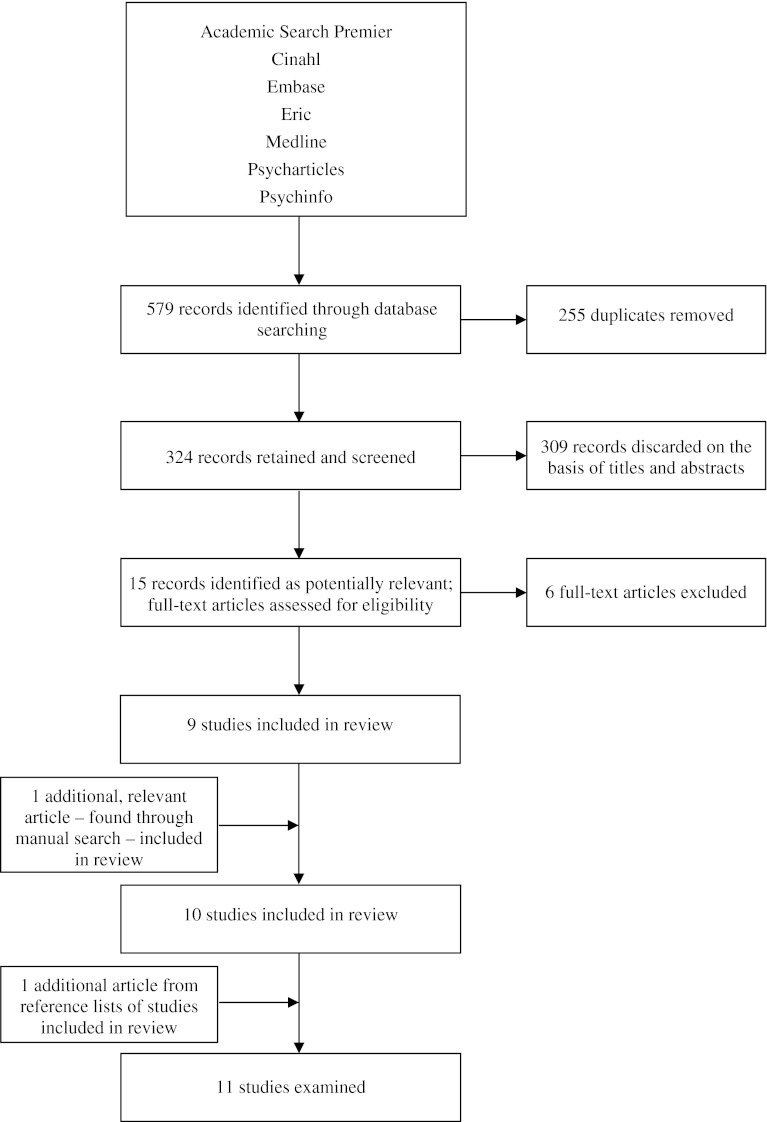
Flowchart of search and selection strategy

The electronic search yielded 579 records. The individual databases yielded 162 (Academic Search Premier), 33 (CINAHL), 167 (EMBASE), 29 (ERIC), 105 (MEDLINE), 0 (PsycARTICLES) and 83 records (PsycINFO) respectively. After removing duplicates, 324 records were left for screening (see Fig. 1). Of these, 309 records were discarded as they did not meet the inclusion criteria. Then the full-text articles of the remaining 15 records were assessed for eligibility. Six studies were excluded either because they concerned an abstract, were written in a non-English language, or because the focus of the questionnaire was too narrow or not purely on educational environment. Our manual search yielded one additional, relevant study. A search of the reference lists of the 10 studies resulting from our search yielded one additional instrument that we included for its relevance.

### Overview of medical educational environment instruments

Inspection of the scales of the 11 included medical educational environment instruments reveals that the instruments demonstrate several similarities (see Table 1). For instance, teaching, supervision and training are recurring themes, as are perceptions of atmosphere, emotional climate and social support. The same is true for perceptions of learning opportunities, orientation to learning, workload, goal direction and emphasis on scholarship. Nevertheless, the instruments also display many differences, for instance, in numbers of scales, which range from three to twelve. These differences may partly be ascribed to the fact that some instruments combine several concepts in one scale, whereas others measure these concepts separately. For example, the Surgical Theatre Educational Environment Measure (STEEM; Cassar 2004) and the Anaesthetic Theatre Educational Environment Measure (ATEEM; Holt and Roff 2004) measure perceptions of workload, supervision and support in combination, whereas the Diagnostic Radiology Clinical Learning Environment Questionnaire (DR-CLE; Bloomfield and Subramaniam 2008), the Dutch Residency Educational Climate Test (D-RECT; Boor et al. 2011), the Postgraduate Hospital Educational Environment Measure (PHEEM; Roff et al. 2005) and the Survey of Learning in Hospital Settings (SLHS; Rotem et al. 1995) measure one or more of these concepts using separate scales. In the same way, the Practice-based Educational Environment Measure (PEEM; Mulrooney 2005) combines perceptions of teaching and learning, whereas the DR-CLE (Bloomfield and Subramaniam 2008) measures these aspects separately. Closer inspection on item level reveals another type of dissimilarity: different instruments use similar items to measure different concepts:

**Table 1:** Brief summary of medical educational environment instruments included in review

Authors	Instrument	Number of items	Scales (*N* items)
Bloomfield and Subramaniam (2008)	DR-CLE questionnaire—diagnostic radiology clinical learning environment questionnaire	24	Supervision (6) Social atmosphere (6) Work-based learning (6) Formal training programmes (3) Workload (3)
Boor et al. (2011)	D-RECT—Dutch residency educational climate test	50	Supervision (3) Coaching and assessment (8) Feedback (3) Teamwork (4) Peer collaboration (3) Professional relations between attendings (3) Work is adapted to residents’ competence (4) Attendings’ role (8) Formal education (4) Role of the specialty tutor (6) Patient sign out (4)
Cassar (2004)	STEEM—surgical theatre educational environment measure	40	Perceptions of trainer and training (13) Perceptions of learning opportunities (11) Perceptions of the atmosphere in the operating theatre (8) Perceptions on supervision, workload and support (8)
Holt and Roff (2004)	ATEEM—the anaesthetic theatre educational environment measure	40	Autonomy (8) Perceptions of atmosphere (10) Workload/supervision/support (7) Perceptions of teaching and teachers (5) Learning opportunities and orientation to learning (10)
Marshall (1978)	MSLES—medical school learning environment survey	50	Breadth of interest Student interaction Organization (goal direction) Flexibility (authoritarianism) Meaningful learning experience Emotional climate Nurturance
Mulrooney (2005)	PEEM—practice-based educational environment measure	37	The practice job (14) GP trainer (10) Teaching and learning (10) Interaction with other health professionals (3)
Roff et al. (2005)	PHEEM—postgraduate hospital educational environment measure	40	Perceptions of role autonomy (14) Perceptions of teaching (15) Perceptions of social support (11)
Roff et al. (1997)	DREEM—Dundee ready education environment measure	50	Perceptions of teaching (12) Perceptions of teachers (11) Academic self-perceptions (8) Perceptions of atmosphere (12) Social self-perceptions (7)
Rotem et al. (1995)	SLHS—survey of learning in hospital settings	46	Autonomy Supervision Social support Workload Role clarity Variety Orientation to learning and teaching Orientation to general practice
Rothman and Ayoade (1970)	LEQ—medical school learning environment questionnaire	65	Evaluative Academic enthusiasm Goal direction Authoritarianism Breadth of interest Student interaction Intellectual maturity
Wakeford (1981)	MSEQ—medical school’s environment questionnaire	49	Friendly Emphasis on concepts (not detail) Emphasis on scholarship Emphasis on ethical aspects Intensive Vocational (vs. ‘scientific’) bias Student involvement (in curriculum etc.) Administratively flexible Educationally facilitative Emphasis on extra-curricular activities Emphasis on written work Enjoyable

- items about good relationships with the teacher are used to measure *perceptions of the trainer* as well as *perceptions of atmosphere* (Mulrooney 2005; Holt and Roff 2004).
- items on workload are used to measure perceptions of *workload/supervision/support* and *perceptions of role autonomy* (Holt and Roff 2004; Roff et al. 2005).
- items on learning opportunities are used to measure *perceptions of learning opportunities* as well as *perceptions of role autonomy* (Cassar 2004; Roff et al. 2005).
- items on learning objectives are used to measure *perceptions of role clarity* and *perceptions of teaching* (Rotem et al. 1995; Roff et al. 1997).

### In-depth search for theoretical frameworks

**Fig. 2:**
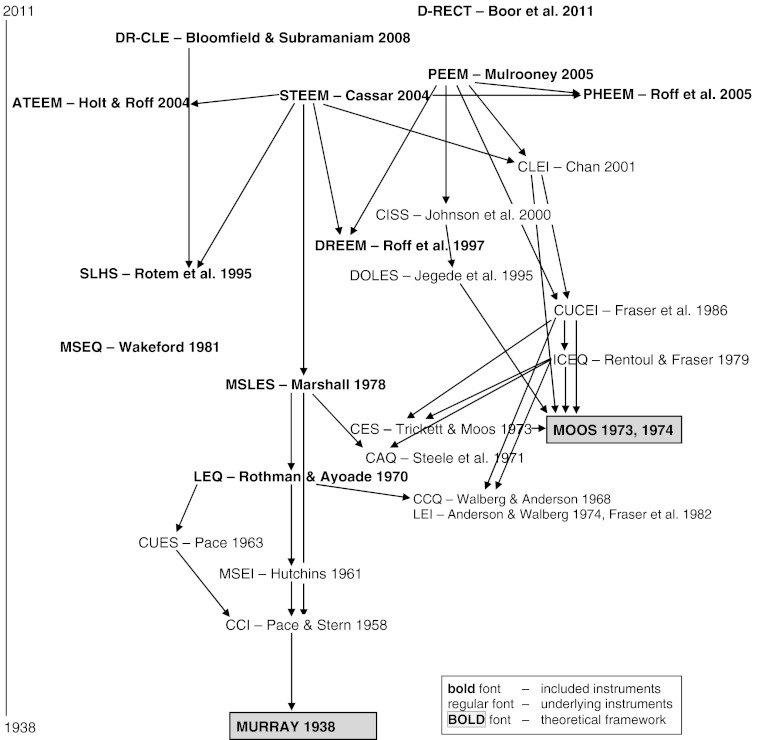
Overview of foundations of education environment instruments included in the study. Underlying instruments not purely focusing on the educational environment or only concentrating on one or some specific aspects of the environment were omitted

Figure 2 illustrates the process of investigating instruments used in the development of the medical educational environment instruments. The most recently developed instruments are positioned at the top of the scheme, with the 11 medical educational environment instruments that formed the start of our in-depth study in bold typeface. Any underlying educational environment instruments are indicated with arrows. The STEEM (Cassar 2004), for instance, was developed using the Clinical Learning Environment Inventory (CLEI; Chan 2001) which was in turn based on the College and University Classroom Environment Inventory (CUCEI; Fraser et al. 1986). Theoretical frameworks that were used in development processes are highlighted in boxes. For example, the theoretical framework of Moos was used to guide the construction of the CLEI (Chan 2001), the CUCEI (Fraser et al. 1986) and the Individualised Classroom Environment Questionnaire (Rentoul and Fraser 1979).

Our in-depth investigation yielded two theoretical frameworks. The first framework was the work of Murray (1938). Murray initially focused on formulating a conceptual scheme for describing personality. When he realized that behavior can not only be attributed to an individual’s personality, but also to the person’s perceptions of the environment, he also focused on the environment. He attempted to operationalize person and environment concepts in commensurate terms. However, Murray casted doubt on his own results. He realized that his criteria for formulating these concepts were not univocal and his scheme was imperfect: the criteria for setting up the categories were not unequivocal (p. 716) and resulted in no more than ‘a rough, preliminary plan to guide perception and interpretation’ (p. 143) (Murray 1938). In addition, the long lists of variables resulting from his efforts are described as rather unstructured and Murray’s work as not offering a systematic theory nor central findings (McAdams 2008). Therefore, we decided not to test the applicability of this framework for evaluating the medical educational environment.

The second theoretical framework that we found was the framework formulated by Moos (1973, 1974). According to Moos, each human environment—irrespective of the type of setting (e.g. psychiatric ward, correctional institution, military training, classroom, therapeutic group, work environment or family setting)—can be described by common sets of dimensions. Moos conceptualized these sets of dimensions in three broad domains:

1. *Personal development* or *goal direction dimensions*, which relate to the basic goals of the specific environment—they assess the basic directions along which personal growth and self-enhancement tend to occur. In educational settings, this domain pertains to achieving the aims of education. An educational environment scoring high on the goal direction domain is characterized by clarity about learning objectives, relevant learning content and constructive criticism.
2. *Relationship dimensions*, which assess the extent to which people are involved in the setting, support and help each other and express themselves spontaneously, freely and openly. A favourable relationship domain is characterized by open communication, friendliness, social and interpersonal support, cohesion and feelings of group spirit. Dimensions representative of positive relationships in educational settings are student involvement, affiliation, (emotional) support and teacher support.
3. *System maintenance and system change dimensions*, which measure the extent to which the environment is orderly and clear in its expectations, maintains control, and responds to change. Examples of the basic dimensions representative of this domain in educational settings are order, organization, rule clarity, teacher control, student influence and innovation. Since the clinical learning environment is part of a work setting, work pressure and physical comfort—a dimension that is, in work settings, representative of this domain—may also be relevant.

Given that Moos’ theoretical framework has been validated in different contexts, including education, we chose to test the applicability of this framework for the medical educational environment.

*Study 2: Applicability of the theoretical framework*

## Methods

To find out whether Moos’ theoretical framework is applicable to the medical educational environment, we investigated whether the items of medical education environment instruments could be mapped into it. Since we did not succeed in obtaining the Medical School Learning Environment Survey (MSLES; Marshall 1978) and the Medical School Learning Environment Questionnaire (LEQ; Rothman and Ayoade 1970), we focused our content analysis on the DR-CLE questionnaire (Bloomfield and Subramaniam 2008), the D-RECT (Boor et al. 2011), the STEEM (Cassar 2004), the ATEEM (Holt and Roff 2004), the PEEM (Mulrooney 2005), Dundee Ready Education Environment Measure (DREEM; Roff et al. 1997), the PHEEM (Roff et al. 2005), the SLHS (Rotem et al. 1995), and the Medical School’s Environment Questionnaire (MSEQ; Wakeford 1981).

### Participants and procedure

Nine researchers of medical education participated in this study. Each of them worked at a medical educational department and they were all involved in curriculum development, teaching, and research of education. They independently ascertained whether the items corresponded with Moos’ theoretical framework. The instruction was to indicate to which of the three domains each item was most applicable. If the participants considered an item as not apt for measuring any of these domains, they could check a box “none of them”. If at least six participants assigned an item to the same domain, we considered the overtone of the item clear and allocated the item to that domain.

## Results

About 94% of the items related to Moos’ framework. Of the total number of 374 items, 256 (68%) were allocated to one single domain, 94 (25%) matched with more than one domain and 24 (6%) could not be mapped into it either because they did not clearly pertain to environment or because the purposes of the items in question were unclear to the raters. Examples of items allocated to one single domain are “I feel I am being well prepared for my profession” and “I am encouraged to develop autonomy in my work here”. These items were assigned to the *goal direction* domain and pertain to the contents and aims of education. Examples of items assigned to the relationship domain are “I feel part of the team”, “I feel comfortable discussing problems in this job”, and “there is a sense of cooperation and mutual respect in the department”. These items all relate to a friendly atmosphere. Examples of the *system maintenance and system change* domain are: “there is a clearly defined pathway to address problems”, “I have protected educational time in this post”, and “my workload in this job is fine”. These items concern organizational or regulative aspects of the environment.

An example of an item that related to more than one domain is “I have opportunities to acquire the skills appropriate to my level of learning”. This item concerns the first domain (the aim of education) as well as the third (the organization in terms of offering enough opportunities) and, hence, could not clearly be allocated to one single domain. Accordingly, half of the raters assigned this item to the first domain and the other half assigned it to the third domain.

An example of the items that could not be classified at all is “I am too tired to enjoy the course”. The cause of the tiredness mentioned in this item may lie outside the educational environment. Therefore, this item does not clearly pertain to educational environment.

## Discussion

The aims of this study were (1) to find a comprehensive theoretical framework that outlines the key concepts that should be measured in ascertaining the quality of the educational environment, and (2) to test the applicability of this framework. We hoped to find out which concepts are essential to the quality of the educational environment and whether medical educational environment instruments measure these concepts. Although, in general, medical educational environment instruments lack solid, established theoretical frameworks, our snowballing method led to a framework that seems sensible and useful for formulating a theoretical framework tailored to the (medical) educational environment (Moos 1973, 1974). Moos’ framework defines three domains as the key elements of human environments: personal development or goal direction, relationship, and system maintenance and system change dimensions. This framework has been validated in different contexts, including education.

Our second study showed that the great majority of the items of nine contemporary medical educational environment instruments could be mapped unto Moos’ framework. Two-thirds of the items were allocated straight to one of the domains and a quarter pertained to more than one domain. Closer inspection of our results showed that in the medical educational environment context, the contents of the three domains relate to *goal orientation* (the content and aims of education), *relationships* (an open and friendly atmosphere and affiliation) and *organization/regulation*. This trichotomy—goal orientation, relationships and organization/regulation—may be valuable as a theoretical framework for (current and future) medical educational environment measures.

The fact that a huge number of items could be mapped unto the framework supports the validity and comprehensiveness of Moos’ general theoretical framework for characterizing human environments. The outcomes of our second study indicate that tailoring this framework to our context implies that evaluating the quality of medical educational environments requires assessment of *goal orientation, relationships* and *organization/regulation* in the environment. Structuring the contents of instruments into these domains may benefit the quality of medical educational instruments. Our study showed that part of the items could be assigned to more than one domain. It is not surprising that not all items could be allocated straight to a single domain, since they were not constructed with the framework in mind. Focusing these items unambiguously on only one of the three domains may contribute to forming coherent scales, which may, in turn, enhance the reliability and validity of the instrument in question.

The validity and comprehensiveness of the framework that we found is supported by previous research. First, Moos found these three domains in nine vastly distinct kinds of settings (Moos 1974). Second, a similar framework was applied in organizational research into the influence of climate on attitude, satisfaction and performance in an organizational setting (Ostroff 1993). This framework was developed independently from Moos and comprises three broad categories of climate perceptions, namely perceptions of the affective (interpersonal and social relations), cognitive (e.g. growth and autonomy) and instrumental aspects (e.g. hierarchy, structure) of the organizational climate. It was commended as a comprehensive classification of organizational climate perceptions that reflects the integration of existing literature (Carr et al. 2003). Third, qualitative studies yielded similar classifications. For example, a study of students’ perceptions of the theatre learning environment yielded three domains as important for successful learning: *educational task* (which pertains to the content and aims of education), *social relations*, and *physical environment and emotional impact* *of the work* (Lyon 2003). A study by Dornan et al. (2005)—focused on the conditions essential for optimizing learning in the clinical environment—revealed three kinds of teacher support (pedagogic, affective and organisational) that also correspond closely with the three domains of educational environment that we identified.

A possible limitation of our study is that we did not statistically (factor) analyse whether the items of the medical educational environment instruments fit the framework. Future research should focus on validating the theoretical framework with quantitative data. To ascertain the value and practical usefulness of the framework, further validation studies should examine whether students’ perceptions of their educational environment (in terms of the three domains) are related to student involvement, satisfaction and achievement. In addition, the outcomes of these studies should be compared to findings of similar research using the original scales.

A second limitation of our study might be the quality of the instruments used in our study. Despite the fact that these instruments were not based on any theoretical framework, we are of the opinion that they constituted a useful point of departure for our in-depth search for a theoretical framework. They were developed carefully by applying thorough qualitative research methods, in several instances even different qualitative research approaches concurrently, like grounded theory (using focus groups and/or Delphi panels), reviewing literature and/or using existing instruments. Besides, they were published in peer-reviewed journals, which represents an acknowledgement of their quality. Last but not least, their wide coverage of environment aspects makes them suitable as an adequate basis for judging the validity of any theoretical frameworks found.

The theoretical framework that we propose for the educational environment is based on Moos (1973, 1974). We realize that Moos’ theory has been developed several decades ago. In the meantime, medical education is broadened with sociocultural perspectives, like situated learning and communities of practice, which may be relevant for measuring educational environment quality (Van der Zwet et al. 2011). Sociocultural theory is considered as a promising and powerful theory that may be valuable for explaining how learning occurs in dynamic contexts like clinical educational environments (Bleakley 2006). Characteristic for sociocultural theory is that the interaction and collaboration with others is acknowledged as influencing students’ learning processes, both through learning knowledge and skills from others, and through becoming familiar with the norms, cultural beliefs and attitudes existing in the communities to which they (the students) are being introduced. The emphasis on interaction and collaboration with others implies that (interpersonal) relationships—belonging to the second domain—are important for students’ learning processes. In addition, learning how to collaborate with others is an important goal to achieve which is related to the first domain, goal direction. Furthermore, the importance that sociocultural theory attaches to interaction and collaboration with others may also have implications for the way education and learning is organized and/or regulated. In our opinion, the proposed framework for measuring medical educational environment quality allows for incorporating these sociocultural perspectives. We noted that, from all the instruments included in our study, the D-RECT (Boor et al. 2011) contains most items representing sociocultural aspects. We also found that all these items could be related to our framework. Given these findings, we think that the proposed theoretical framework and sociocultural theory may supplement each other and thus help to carry the medical education field forward. We also think that the proposed framework may add to the understanding of the functioning and effectiveness of situated learning and communities of practice (Lave and Wenger 1991; Dornan et al. 2007; Fuller et al. 2005; Ellström 2001).

The current theoretical framework may also add to other, related fields that are important to the quality of students’ learning processes. It may, for instance, add to the understanding of the functioning and effectiveness of supervision: in an extensive literature review Kilminster and Jolly (2000) highlight *supervision relationship*, *feedback*, and *trainee control over the supervisory process* and *finding sufficient time for supervision* as the most important features of effective supervision. These key features clearly correspond with the domains relationships, goal orientation, and organization/regulation, respectively. In a similar way, the framework may add to the understanding of roles and quality of clinical teachers and to the improvement of (clinical) teaching (Harden and Crosby 2000; Ramani and Leinster 2008; Skeff 1988; Fluit et al. 2010).

The merits of the proposed theoretical framework are that it clearly delineates three distinct educational environment domains, or—in other words—three distinct sets of common educational environment dimensions. It ensures coverage of the essential components of the environment and, at the same time, enables educators in practice to tailor evaluations to specific settings. The categorization into *goal orientation*, *relationships* and *organization/regulation* may help educators restructure existing instruments or develop new ones in such a way that they cover the entire educational environment adequately. In addition, the framework enables multi-site research on a conceptual level: if, for example, relationships turn out to be the educational environment domain which influences student motivation and achievement most, similar outcomes should be found in different settings while using different instruments. In the same way, the framework—if commonly applied—enables educators to compare the quality of their own educational environment with that of others, even if these were evaluated using different instruments. We would even go so far as to state that tailoring items to the own setting using this theoretical framework may be a better approach to evaluation than translating and back translating instruments, since the latter bears the risk of including irrelevant cultural or contextual aspects.

In conclusion, we found a universally applicable set of domains that seems to cover the entire educational environment and comprise the essential concepts: *goal orientation*, *relationships* and *organization/regulation*. This theoretical framework seems valuable for research into the quality of medical educational environments and for constructing tools for assessing the medical educational environment. Therefore, we recommend this framework as a theoretical underpinning of medical educational environment measures. Ultimately, applying this framework may help to create educational environments that are conducive to learning. We hope that our study inspires educators to incorporate this framework into daily practice and research. Furthermore, we challenge researchers to test the framework with existing or new instruments. Their findings may help to achieve evidence-based practice on how to ascertain the quality of educational environment best and move the educational research field forward (Prideaux and Bligh 2002; Eva 2008; Eva and Lingard 2008; Bordage 2009).
